# Supplementary material for: Phenologs at knowledge-graph scale: a mouse-to-slime-mold catalog of candidate genes for thousands of human diseases
Source: bioRxiv. 2026 Oct 27:2026.10.23.761438. Preprint. [Version 1] doi: 10.64898/2026.10.23.761438 (PMC13733769; doi:10.64898/2026.10.23.761438)
Supplement: Supplementary file 1 [file NIHPP2026.10.23.761438V1-supplement-1.pdf]

bioRxiv preprint doi: https://doi.org/10.64898/2026.10.23.761438; this version posted October 27, 2026. The copyright holder for this preprint (which was not certified by peer review) is the author/funder, who has granted bioRxiv a license to display the preprint in perpetuity. It is made available under a CC-BY 4.0 International license.

Thomas, Paul D., Dustin Ebert, Anushya Muruganujan, Tremayne Mushayahama, Laurent-Philippe Albou, and Huaiyu Mi. 2022. "PANTHER: Making Genome-Scale Phylogenetics Accessible to All." *Protein Science* 31 (1): 8–22.

Unni, Deepak R., Sierra A. T. Moxon, Michael Bada, et al. 2022. "Biolink Model: A Universal Schema for Knowledge Graphs in Clinical, Biomedical, and Translational Science." *Clinical and Translational Science* 15 (8): 1848–1855.

Vasilevsky, Nicole A., Sabrina Toro, Nicolas Matentzoglu, et al. 2025. "Mondo: Integrating Disease Terminology Across Communities." *Genetics*, October 6, iyaf215.

Woods, John O., Ulf Martin Singh-Blom, Jon M. Laurent, Kriston L. McGary, and Edward M. Marcotte. 2013. "Prediction of Gene-Phenotype Associations in Humans, Mice, and Plants Using Phenologs." *BMC Bioinformatics* 14 (June): 203.

# Supplementary Information

## Supplementary Note 1. Monarch KG filtering

The Biolink Schema is used by the Monarch KG as an underlying data model representation. We identified the relevant sets of nodes and edges for the phenologs calculations we wanted to perform and created a subgraph of the larger Monarch KG to begin our analysis.

Table S1. Monarch KG Biolink Categories, Associations, and Predicates

| Kind | Biolink category | Predicate |
|---|---|---|
| Node | biolink:Gene | n/a |
| Node | biolink:PhenotypicFeature | n/a |
| Node | biolink:Disease | n/a |
| Edge | biolink:GeneToPhenotypicFeatureAssociation | biolink:has_phenotype |
| Edge | biolink:CausalGeneToDiseaseAssociation | biolink:causes; biolink:associated_with_increased_likelihood_of |
| Edge | biolink:CorrelatedGeneToDiseaseAssociation | biolink:gene_associated_with_condition; biolink:contributes_to |
| Edge | biolink:GeneToGeneHomologyAssociation | biolink:orthologous_to |

**Table S1. Monarch KG node and edge types selected for.** The Biolink node and edge types we select for are listed within the table for our analysis. Note that GeneToGeneHomology edges were restricted to protein coding gene pairs where a PANTHER protein family classification is given as evidence within the edge provenance.

Additionally, each species within the knowledge graph uses its own ontologies for phenotype and or disease node name representation (**Table S2**). Depending on the structure of the ontology used, there may or may not be terms needed to be excluded from our analysis (we are

bioRxiv preprint doi: https://doi.org/10.64898/2026.10.23.761438; this version posted October 27, 2026. The copyright holder for this preprint (which was not certified by peer review) is the author/funder, who has granted bioRxiv a license to display the preprint in perpetuity. It is made available under a CC-BY 4.0 International license.

primarily interested in only abnormal phenotypes). To achieve our filtering goals of only including gene-phenotype associations where an alteration of the “gene” is causing or likely causing an abnormal phenotype, we precompute sets of nodes from each ontology to either include or exclude from our initial subgraph building process. We reconstruct each species' individual ontology from phenio, a larger overarching ontology linking many ontologies together that the Monarch KG leverages under the hood in each release (Putman et al. 2023). This allows us to achieve version parity with all ontologies used within a single extra file, rather than downloading each ontology individually (https://github.com/monarch-initiative/phenio/releases/latest/download/phenio-relation-graph.gz). Each ontology’s node/edge structure can then be reconstructed by following the directed graph from the edges linking between intra ontology nodes based on the prefix identifiers (e.g. HP, MP, MONDO). Filtering can then be performed based on whether or not a term descends from a chosen root term (**Table S3**). For example, fission yeast in our calculations only includes phenotype terms that are descendants of the “abnormal phenotype” term within the Fission Yeast Phenotype Ontology (FYPO) (Harris et al. 2013).

Table S2. Species disease and phenotype ontologies

| Taxon label | Phenotype ontology | Disease ontology | Phenotype prefix | Disease prefix |
|---|---|---|---|---|
| *Danio rerio* | Zebrafish Phenotype Ontology | n/a | ZP | n/a |
| *Xenopus tropicalis* | Xenopus Phenotype Ontology | n/a | XPO | n/a |
| *Caenorhabditis elegans* | C. elegans Phenotype Ontology | n/a | WBPhenotype | n/a |
| *Rattus norvegicus* | The Mammalian Phenotype Ontology | n/a | MP | n/a |
| *Mus musculus* | The Mammalian Phenotype Ontology | n/a | MP | n/a |
| *Schizosaccharomyces pombe* | Fission Yeast Phenotype Ontology | n/a | FYPO | n/a |
| *Dictyostelium discoideum* | Dicty Phenotype Ontology | n/a | DDPHENO | n/a |
| *Homo sapiens* | Human Phenotype Ontology | Mondo | HP | MONDO |

**Table S2. Ontologies used for each species within the Monarch KG.** We select specifically for nodes with these prefixes within the subgraph building process, along with the relevant biolink model category. For example, a node with an ID beginning with “ZP:” would also need to coincide with the biolink category PhenotypicFeature.

Table S3. Ontology term filtering

| Ontology | Terms included |
|---|---|
| Mondo | Only "human disease" terms (Include everything under MONDO:0700096) |
| Human Phenotype Ontology | Only "phenotypic abnormality" terms (Include everything under HP:0000118) |
| Fission Yeast Phenotype Ontology | Only "abnormal phenotype" terms (Include everything under FYPO:0001985) |

bioRxiv preprint doi: https://doi.org/10.64898/2026.10.23.761438; this version posted October 27, 2026. The copyright holder for this preprint (which was not certified by peer review) is the author/funder, who has granted bioRxiv a license to display the preprint in perpetuity. It is made available under a CC-BY 4.0 International license.

| Ontology | Terms included |
|---|---|
| The Mammalian Phenotype Ontology | Everything except for "normal phenotype" terms (Exclude everything under MP:0002873) |
| Dicty Phenotype Ontology | Everything except for "wild type" terms (Exclude everything under DDPHENO:0000142) |

**Table S3. Phenotype and disease term filtering via ontological structure.** Not all phenotypes are necessarily valid for this study and thus needed to be avoided when creating the initial subgraph of the original KG. We select for abnormal phenotypes, and human disease specifically when it is needed. Each ontology was examined visually using EMBL-EBI Ontology Lookup Service (McLaughlin et al. 2025) in order to identify the proper terms.

## Supplementary Note 2. Gene homology

The primary source of gene homology information within the Monarch KG originates from PantherDB's (Thomas et al. 2022) "AllOrthologs.tar.gz", which catalogs all orthologous relationships between the gene sets of more than 140 different species. This information is ingested by the Monarch KG for a select set of species as gene-gene orthology relationships. Each gene with an ortholog in at least one other species is assigned a protein family identifier by PantherDB (e.g. PTHR11482). The eight species used in this study are the only species in which gene-phenotype relationships exist within the graph.

As described in (Woods et al. 2013), the direction of comparison between species-specific gene-phenotype networks becomes a computational enigma when homology is treated gene-by-gene, rather than collapsing orthologous and paralogous relationships into "orthogroups" that create a single point of comparison across divergent species gene families. We approximate this using the PantherDB protein family assignment for each gene, collapsing the 19,159 human genes carrying an assignment into 7,489 orthogroups and removing the asymmetry caused by unevenly expanded gene families.

bioRxiv preprint doi: https://doi.org/10.64898/2026.10.23.761438; this version posted October 27, 2026. The copyright holder for this preprint (which was not certified by peer review) is the author/funder, who has granted bioRxiv a license to display the preprint in perpetuity. It is made available under a CC-BY 4.0 International license.

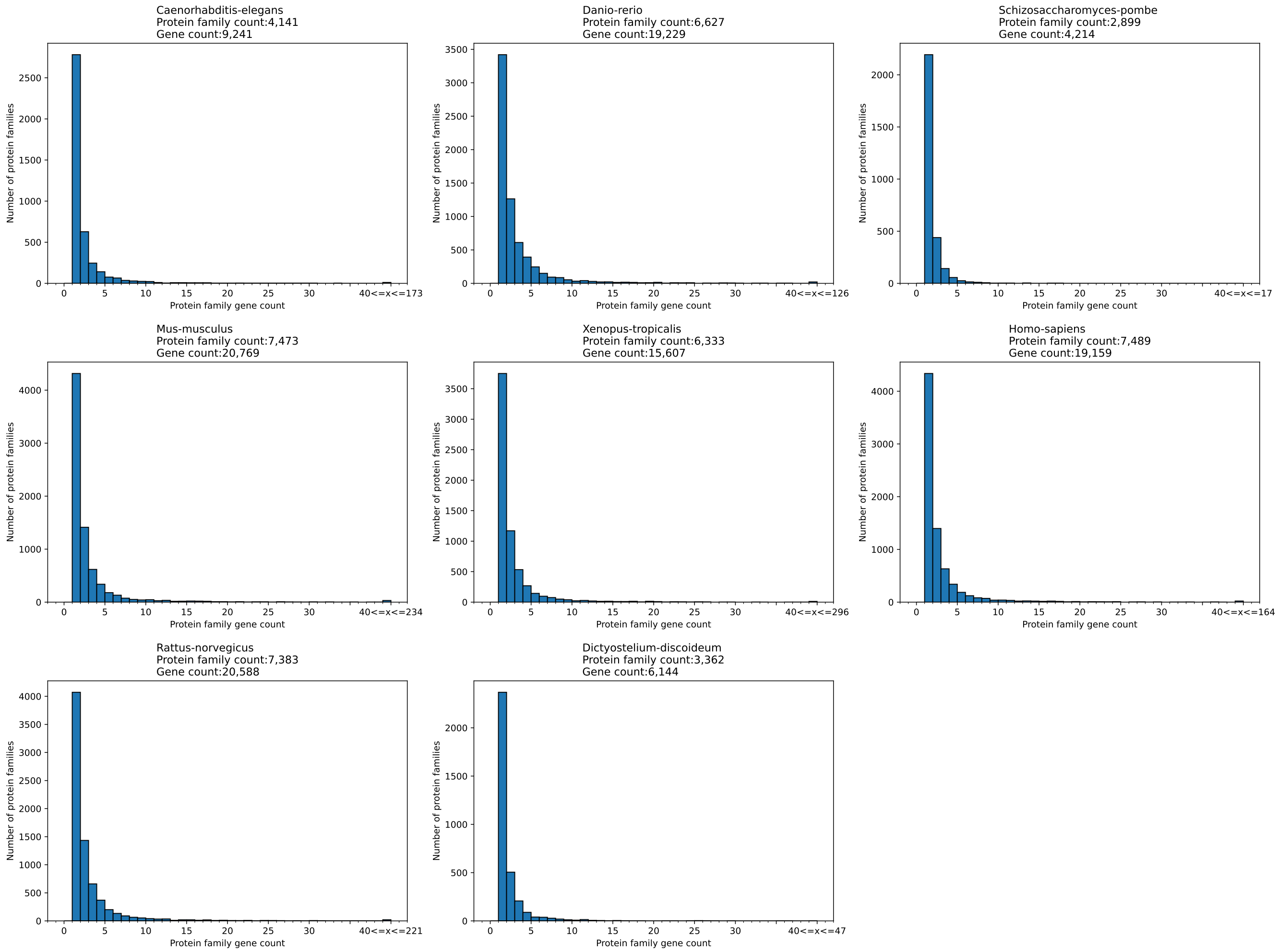


**Figure S1. Distribution of the number of genes per protein family for each species used.** PantherDB protein family gene count distribution for each species. We note this is a construct of the species representation within the Monarch KG in the sense that if a gene does not have an ortholog in another species within our dataset, it is not included. For example, by PantherDB's count there are 19,450 protein coding genes in humans, but our dataset only captures 19,159 (98.5%) of them because of the species diversity limitation within our data and genes unique to humans.

We collapse at PantherDB's family level (e.g. PTHR11482) rather than its finer subfamily subdivisions (e.g. PTHR11482:SF7), because the family level maximizes the shared cross-species members available to each comparison; subfamilies, or requiring a direct one-to-one ortholog edge, would fragment these sets and reduce the statistical power of the initial phenolog test. For this reason the orthogroup abstraction is used only to detect phenologs. However, the underlying gene-level ortholog edges are retained to rank gene candidates downstream. Each family-level signal is resolved back to the specific human ortholog(s) of the driving model gene (42% of human families contain more than one human gene).

The effects of this collapse on human disease nodes, in terms of gene and protein-family annotation counts, are shown in **Fig. S2**. The gene networks remain robust when collapsed to

bioRxiv preprint doi: https://doi.org/10.64898/2026.10.23.761438; this version posted October 27, 2026. The copyright holder for this preprint (which was not certified by peer review) is the author/funder, who has granted bioRxiv a license to display the preprint in perpetuity. It is made available under a CC-BY 4.0 International license.

the orthogroup level: of the 1,116 human diseases annotated with two or more family-bearing genes, only 72 (6.5%) collapse to a single orthogroup, so multi-gene diseases almost always retain more than one point of comparison.

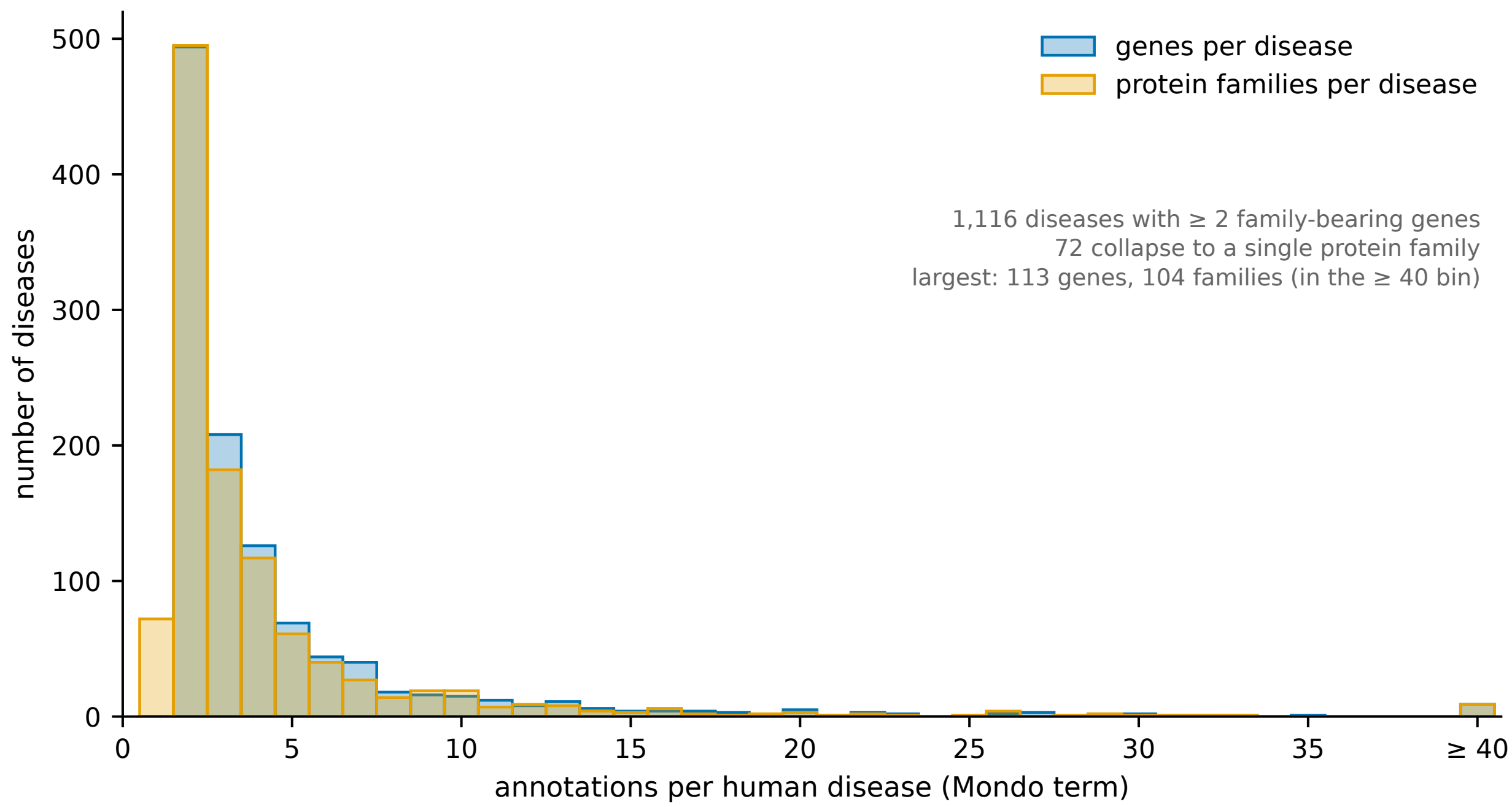


**Figure S2. Distribution of the number of protein families or genes associated with each MONDO disease term.** Gene-disease networks remain stable when collapsed to the orthogroup level, with only 72 disease nodes collapsing down to a single protein family annotation from an original protein family annotation count of ≥ 2.

## Supplementary Note 3. False discovery rate for initial phenolog calculations

The number of phenolog comparisons to be made between any two species corresponds to the number of phenotypes in species-a multiplied by the number of phenotypes in species-b, where each phenotype must be linked to at least one orthologous gene between the two species. The number of initial phenolog calculations between human diseases and model species phenotypes is found in **Table S4**.

Table S4. Human gene-disease comparison between each model species phenotypes

| Human disease (Species A) | Model species (Species B) | Comparable human diseases | Comparable phenotypes | Common orthologs | Pairwise comparisons |
|---|---|---|---|---|---|
| *Homo sapiens* | *Mus musculus* | 7,919 | 10,998 | 7,394 | 87,093,162 |
| *Homo sapiens* | *Danio rerio* | 7,725 | 26,040 | 6,479 | 201,159,000 |
| *Homo sapiens* | *Caenorhabditis elegans* | 5,925 | 1,707 | 3,931 | 10,113,975 |
| *Homo sapiens* | *Schizosaccharomyces pombe* | 3,072 | 3,646 | 2,301 | 11,200,512 |
| *Homo sapiens* | *Rattus norvegicus* | 7,879 | 1,156 | 7,296 | 9,108,124 |

bioRxiv preprint doi: https://doi.org/10.64898/2026.10.23.761438; this version posted October 27, 2026. The copyright holder for this preprint (which was not certified by peer review) is the author/funder, who has granted bioRxiv a license to display the preprint in perpetuity. It is made available under a CC-BY 4.0 International license.

| Human disease (Species A) | Model species (Species B) | Comparable human diseases | Comparable phenotypes | Common orthologs | Pairwise comparisons |
|---|---|---|---|---|---|
| *Homo sapiens* | *Xenopus tropicalis* | 7,522 | 124 | 6,173 | 932,728 |
| *Homo sapiens* | *Dictyostelium discoideum* | 3,719 | 235 | 3,021 | 873,965 |

**Table S4. Number of possible comparisons between human mondo disease terms and model species phenotype terms.**

Given the sufficiently large number of comparisons being made between each pair of species, the false discovery rate correction methodology previously described in (McGary et al. 2010) is used. This method compares the phenologs computed from 1,000 randomized species gene-association networks. Note, that within this network randomization process, for any given gene-association (disease or phenotype), the number of genes originally associated with it will remain the same each trial, and a random set of genes is pulled from the common orthologs found between the two species. For a gene to be available to draw from the random pool, it must also be connected to a phenotype. Each trial computes the set of pairwise comparisons between the two species networks and the hypergeometric p-values are recorded across all comparisons and all trials. To obtain an estimated false discovery rate of 5% we compare the p-value distribution from the real phenolog calculations, to the distribution of the p-values taken from the randomized trials. We find the p-value at which 5% or less of the real data overlaps with the randomized data. In the example given in **Fig. S3**, we show the log scaled histogram of p-values obtained by comparing all human diseases against all mouse phenotype terms (“real”), along with the 1,000 randomized versions of these calculations. Note, we first pool all 1,000 random trials together, create the histogram, then divide the y-values obtained (number of data-points within a given x-axis bin) by 1,000 to set the randomized trial data on the same scale as our real calculations. In this example we find 51,766 real “phenologs” with a p-value ≤ $2.7 \times 10^{-4}$, in which we could expect 3.8% to be false positives given the overlap found with the randomized background distribution. The next higher p-value would push that overlap beyond the allowed 5% so we stop at 3.8%. This becomes our “significance” threshold for determining whether or not to include a disease-phenotype comparison in the set of data used downstream. The resulting number of disease-phenotype comparisons passing each species threshold can be found in **(Fig. 2C)**.

bioRxiv preprint doi: https://doi.org/10.64898/2026.10.23.761438; this version posted October 27, 2026. The copyright holder for this preprint (which was not certified by peer review) is the author/funder, who has granted bioRxiv a license to display the preprint in perpetuity. It is made available under a CC-BY 4.0 International license.

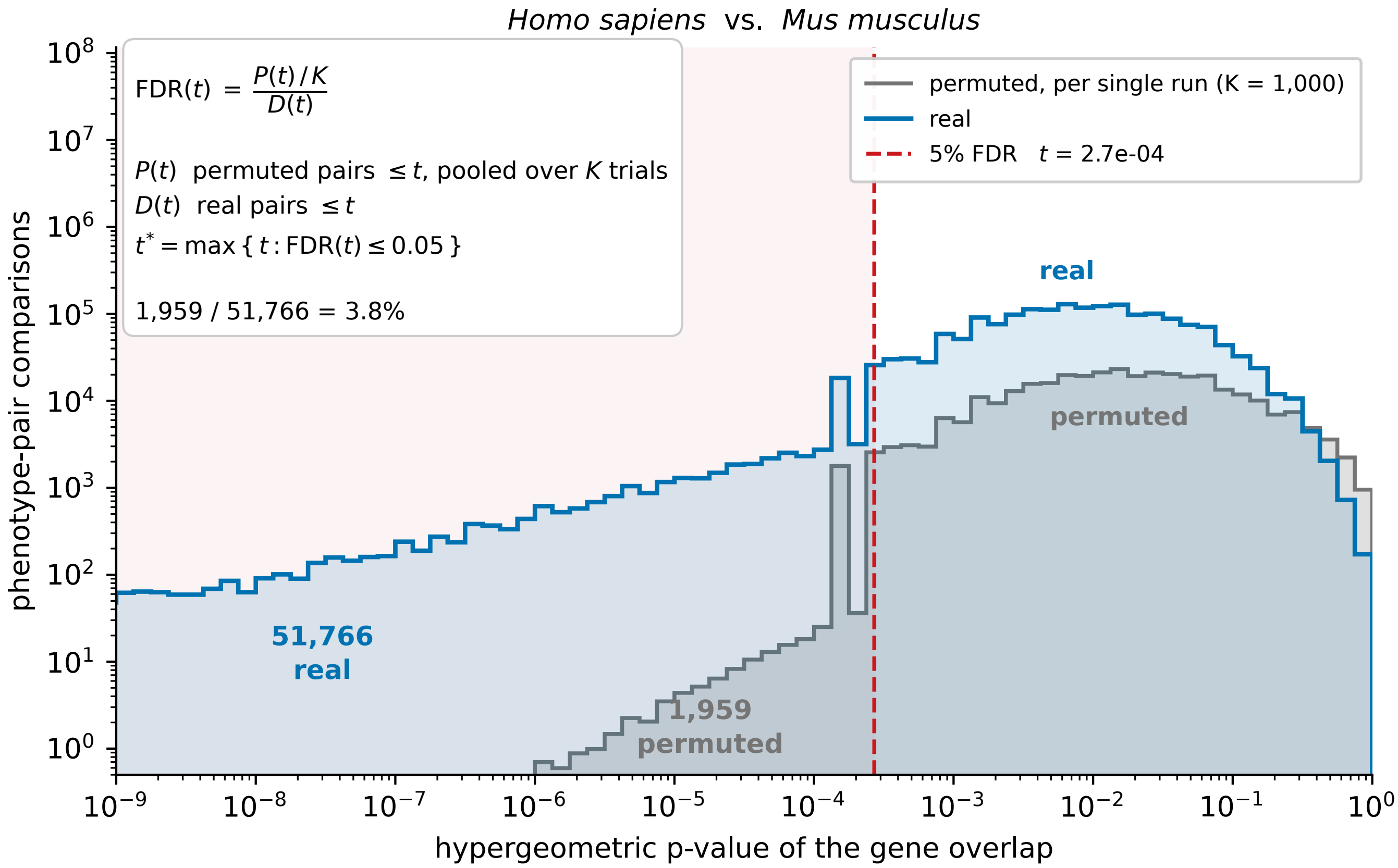


**Figure S3. A visualization of how we compute the cut-off p-value for our false discovery rate corrections.** In this figure we present the distribution of p-values for the disease-phenotype linkages from real data and p-values from one thousand permuted trials between human and mouse. The dashed red line shows the maximum point where our goal of having at least twenty times more real pairs than permuted pairs is achieved (i.e. 5% estimated FDR).

bioRxiv preprint doi: https://doi.org/10.64898/2026.10.23.761438; this version posted October 27, 2026. The copyright holder for this preprint (which was not certified by peer review) is the author/funder, who has granted bioRxiv a license to display the preprint in perpetuity. It is made available under a CC-BY 4.0 International license.

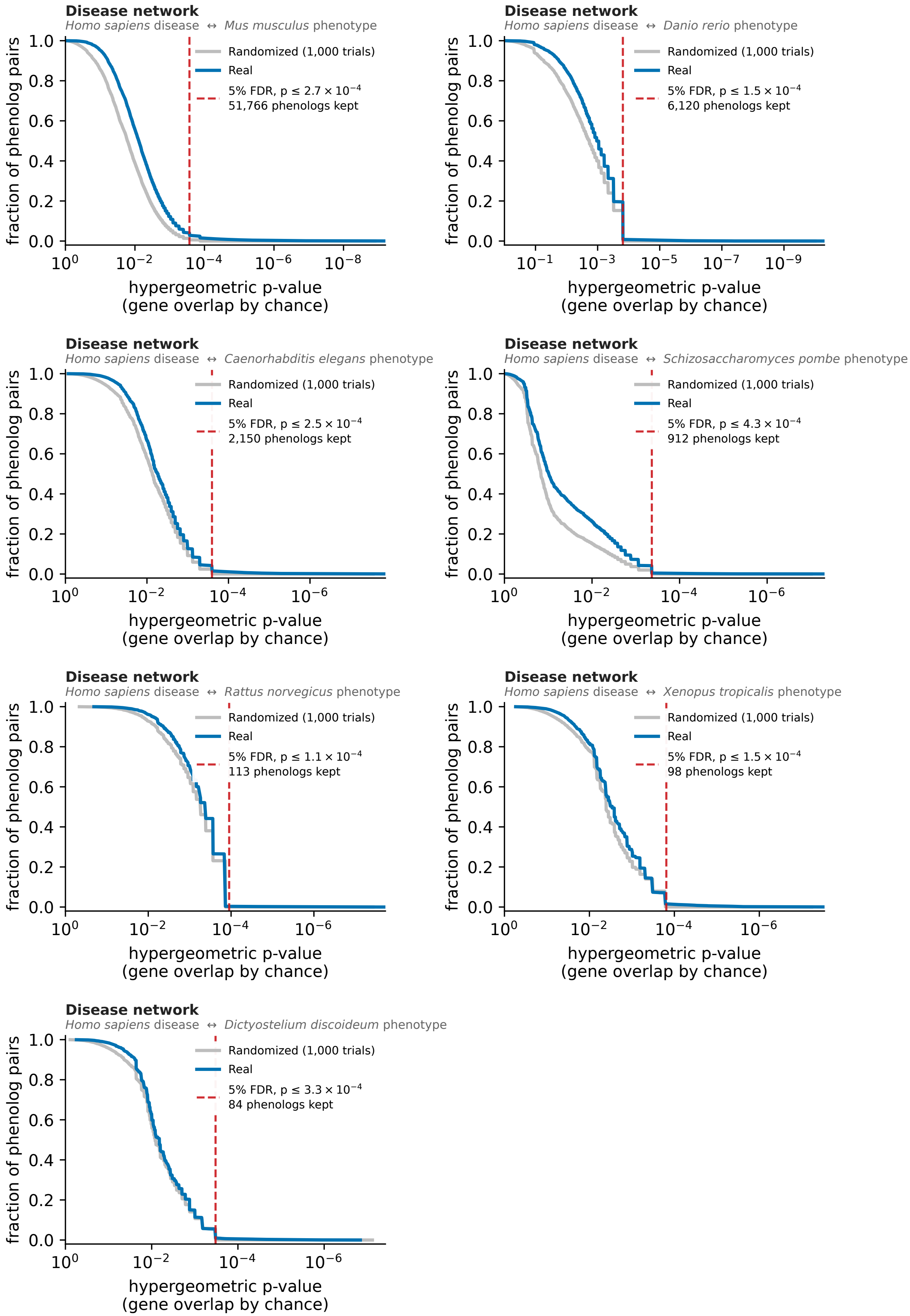

bioRxiv preprint doi: https://doi.org/10.64898/2026.10.23.761438; this version posted October 27, 2026. The copyright holder for this preprint (which was not certified by peer review) is the author/funder, who has granted bioRxiv a license to display the preprint in perpetuity. It is made available under a CC-BY 4.0 International license.

**Figure S4. Phenolog significance determination, Human gene-disease network.** Results of the 1,000 random trials between randomized human gene-disease and model species gene-phenotype networks for each species within our comparison set. Phenolog p-values are pooled across all 1,000 random trials within a given model species comparison, and then sorted (for comparisons where there is ≥ 1 gene/ortholog overlapping). We highlight with the red dashed line the p-values used in this study for each species representing an estimated 5% false discovery rate in accordance with work prior (McGary et al. 2010).

bioRxiv preprint doi: https://doi.org/10.64898/2026.10.23.761438; this version posted October 27, 2026. The copyright holder for this preprint (which was not certified by peer review) is the author/funder, who has granted bioRxiv a license to display the preprint in perpetuity. It is made available under a CC-BY 4.0 International license.

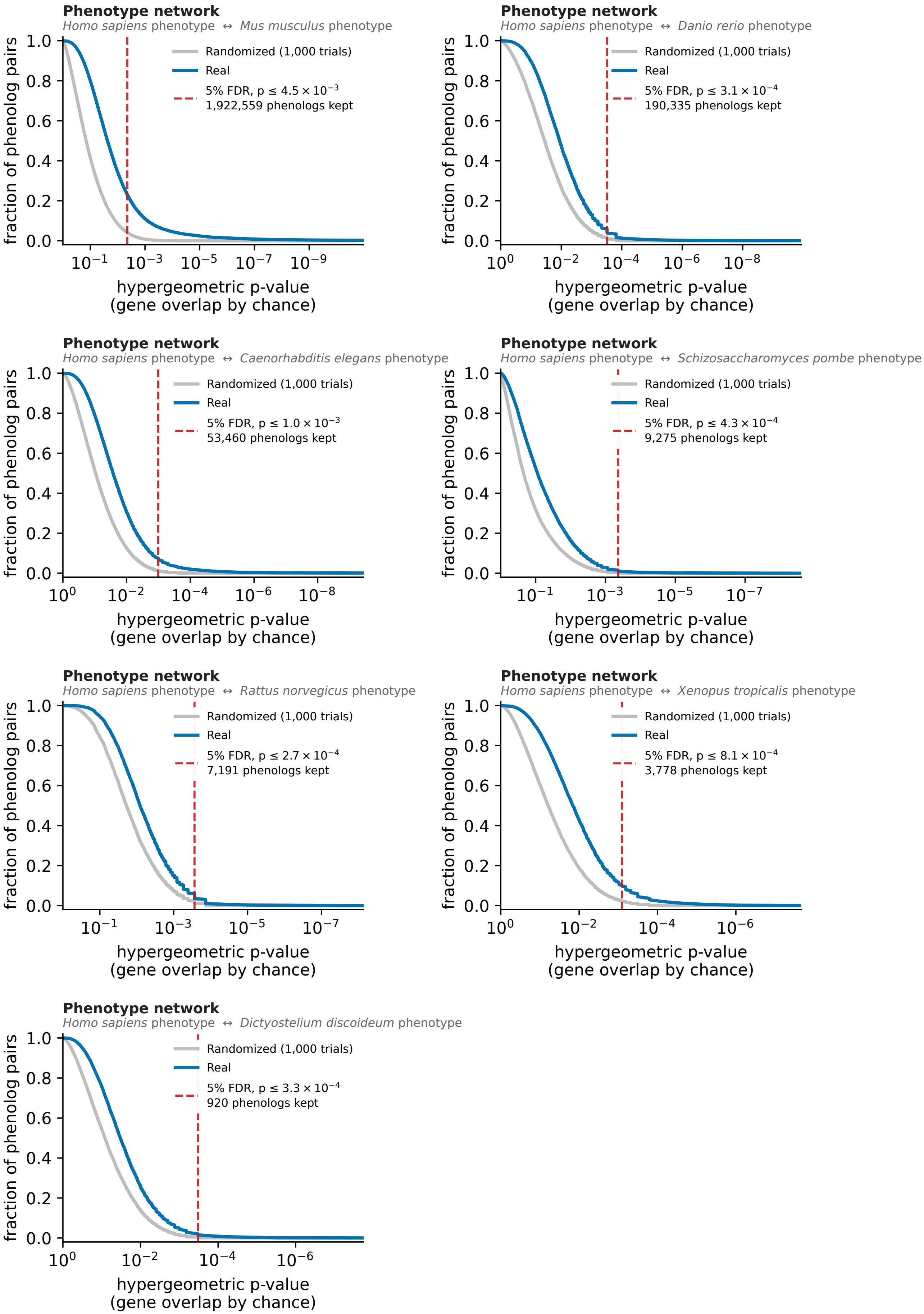

bioRxiv preprint doi: https://doi.org/10.64898/2026.10.23.761438; this version posted October 27, 2026. The copyright holder for this preprint (which was not certified by peer review) is the author/funder, who has granted bioRxiv a license to display the preprint in perpetuity. It is made available under a CC-BY 4.0 International license.

**Figure S5. Phenolog significance determination, Human gene-phenotype network.** Results of the 1,000 random trials between randomized human gene-phenotype and model species gene-phenotype networks for each species within our comparison set. Phenolog p-values are pooled across all 1,000 random trials within a given model species comparison, and then sorted (for comparisons where there is ≥ 1 gene/ortholog overlapping). We highlight with the red dashed line the p-values used in this study for each species representing an estimated 5% false discovery rate in accordance with work prior (McGary et al. 2010)

## Supplementary Note 4. Naive Bayes gene ranking method

To integrate information across multiple "phenologs", for a single human disease, (Woods et al. 2013) used a matrix based formalization to help describe the problem where $X$ is a set of gene-phenotype predictions, and $X_{ij}$ describes a value associating gene $i$ to phenotype $j$. Here, we are using human diseases as the "phenotype" columns, and HGNC identifiers as the "gene" rows. In this way, one is able to rank how strongly any given $gene\ i$ is associated with $disease\ j$ across all genes and diseases. To compute $X_{ij}$ both (McGary et al. 2010) and (Woods et al. 2013) use the following equation, which they describe as the naïve Bayes scheme:

$$\text{(S1)} \qquad X_{ij} = P(gene\ i \in disease\ j \mid k\ phenologs) = 1 - \prod_{l=1}^{k} (1 - f_{ijl} w_{jl})$$

where

$$\text{(S2)} \qquad f_{ijl} = P(gene\ i \in disease\ j \mid phenotypes\ j\ and\ l\ are\ phenologs)$$

$$\text{(S3)} \qquad w_{jl} = P(phenotypes\ j\ and\ l\ are\ phenologs)$$

Here, $w_{jl}$ is 1 - hypergeometric p-value that was used to compute the initial association (McGary et al. 2010). For $f_{ijl}$, we use $v/n$, which is the "fraction of the number of genes common to both phenotypes *j* and *l* over the number of genes known to be involved in phenotype *j*" (Woods et al. 2013). In our case, phenotype $j$ is a human disease, and phenotype $l$ is a cross species phenotype. In this way, we are computing the probability of the phenolog association being true multiplied by the approximate probability of a single gene-disease association already being true for a particular phenolog pairing. Subtracting from 1 then gives the probability of that gene-disease pairing being "false". Multiplying these probabilities together across k phenologs and further subtracting from 1 gives the probability of that gene-disease pairing being true. We will also refer to this method the naive Bayes (nb) metric as previously described.

## Supplementary Note 5. Hypergeometric gene ranking method

We introduce a new method for integrating information across top k-associated phenologs. For this new formulation of the gene-disease association matrix $X$, we define each gene-disease association value by computing the upper-tail probability of the hypergeometric distribution of

bioRxiv preprint doi: https://doi.org/10.64898/2026.10.23.761438; this version posted October 27, 2026. The copyright holder for this preprint (which was not certified by peer review) is the author/funder, who has granted bioRxiv a license to display the preprint in perpetuity. It is made available under a CC-BY 4.0 International license.

the gene's occurrence within a disease's top k phenologs P(X≥c). This is implemented using the survival function in Python's SciPy as,

$$\text{(S4)} \qquad X_{ij} = P(gene\ i\ \in\ disease\ j\,|\,k\ phenologs) = scipy.stats.hypergeom.sf(c-1,\ N,\ m,\ n)$$

where $n$ is the total number of genes found connected to any of the top k ranked phenotypes (phenologs). Or more formally:

$$\text{(S5)} \quad n(k) = \left|\left(x_1,\ x_2,\ ...\ x_k\right)\right|$$

where $x$ is a gene belonging to a phenotype within the top k ranked phenologs. In our metric formulation, $n$ can be thought of as the number of times we draw a gene prediction given our initial pool of candidate phenotypes found to be associated with a disease. The number of times each gene occurs within our background network is $m$. More specifically, $m$ is the number of phenotypes the gene of interest is connected to across all species within our graph. $N$ is the background total number of existing phenotype-gene edges where each gene must have an ortholog in human (or the base species one is using for comparison). The number of times each gene shows up within our top ranked k phenologs is used as $c$. We can then ask what is the probability of gene $i$ showing up >= $c$ times given its background count within the network $m$, $N$ total associations we could potentially draw from, and $n$ genes we originally pulled in from our k top ranked phenologs.

## Supplementary Note 6. Temporal evaluation parameter sweep

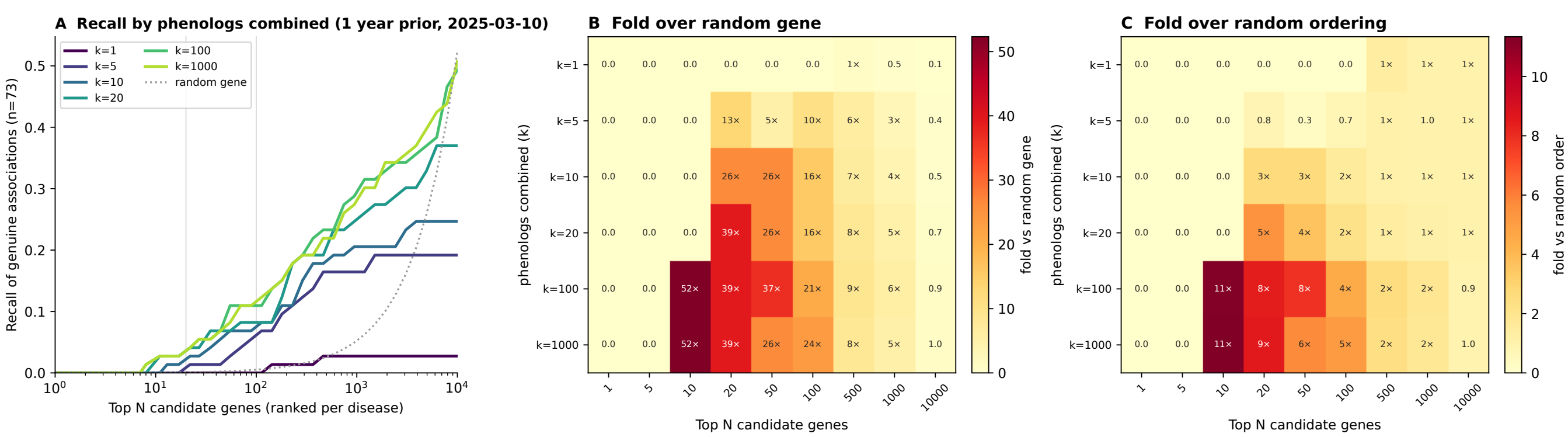


**Figure S6. Parameter sweep of the one-year temporal validation across phenologs combined (k) and shortlist depth (N).** All panels use hg rankings built from Monarch KG release 2025-03-10 and scored against the 73 genuine gene-disease pairs added by release 2026-03-06 (Table 2), as in Fig. 3. (A) Recall of the 73 pairs within each disease's top N candidate genes for k = 1, 5, 10, 20, 100 and 1,000 phenologs combined per disease, one curve per k. The dotted line is the recall expected when N genes are drawn at random from the U human genes carrying an ortholog edge to a modeled species, min(N, U)/U. Faint vertical lines mark N = 20 and N = 100. (B) Fold enrichment over that random-gene expectation for every combination of k (rows) and N (columns); each cell is the recall at N divided by min(N, U)/U, printed in the cell (values of one or more carry a multiplication sign). (C) Fold enrichment over a random ordering of each disease's own candidate list: the recall at N divided by the recall expected if the candidate genes surfaced for a disease were ranked at random, which for a pair whose disease has

bioRxiv preprint doi: https://doi.org/10.64898/2026.10.23.761438; this version posted October 27, 2026. The copyright holder for this preprint (which was not certified by peer review) is the author/funder, who has granted bioRxiv a license to display the preprint in perpetuity. It is made available under a CC-BY 4.0 International license.

M candidates is min(N, M)/M. Panel B measures the gain from restricting a disease to its phenolog-derived candidates and ranking them; panel C isolates the gain from the ranking alone.

The analysis leading to (**Table 2**) identified 73 genuine new gene-disease pairs added to the Monarch KG between releases 2025-03-10 and 2026-03-06, spanning 43 diseases. As in (**Fig. 3A**), we rank each of these diseases' candidate genes from the earlier release and ask where the new genes fall, here across a grid of k, the number of top phenologs combined per disease, and N, the candidate gene list depth (**Fig. S6**). Enrichment over a randomly drawn gene is highest when only considering the top candidates, where recall is still small, so the practical window appears to be roughly the top 20 to 100 candidates, which retains double-digit fold enrichment together with meaningful recall, and enrichment still eightfold or more out to the top 500 (**Fig. S6A,B**). The fold enrichment over a random ordering of each disease's own candidate list is smaller, 11-fold at N = 10 and 4 to 5-fold at N = 100, and is the stricter measure of what the ranking itself adds (**Fig. S6C**). The permutation p-value reported in the main text tests this stricter null, comparing the observed recall at N with 5,000 random orderings of each disease's candidate gene list, so the smallest attainable value is $2 \times 10^{-4}$. Within this window hg and nb are statistically indistinguishable ($p = 0.32$; **Fig. 3A**), as expected given the fact they score from the same pool of surfaced genes. This is also consistent with the parity between nb and hg when ranking candidates within a disease under leave-one-out evaluation (**Supplementary Note 7**).

## Supplementary Note 7. Leave-one-out performance evaluation

We adapt the orthogroup-based leave-one-out cross validation of (Woods et al. 2013) to ask whether known gene-disease associations within the Monarch KG can be recovered once they are hidden from the method, and how highly they rank when they are. For each of the 2,001 PANTHER protein families carrying at least one human disease annotation, we remove the family's genes from the human gene-disease network, recompute the full set of phenologs, and rebuild the gene rankings with both the naive Bayes (nb) and hypergeometric (hg) schemes (Supplementary Notes 4 and 5) at k = 1, 5, 20 and 100 phenologs combined per disease. The whole family is withheld rather than a single gene because a paralog left behind in the same family would otherwise stand in for the hidden gene and make the test trivial. Ranks are then read at the gene level, so a family counts as recovered only through the specific genes that were annotated to the disease. Tied scores receive the average rank, and when a disease has more than one hidden gene we record the median of their ranks.

The resulting ranks are read in two directions (**Fig. S7A,B**). In the first, we take a disease and ask where its hidden genes fall among all candidate genes ranked for that disease (**Fig. S7A**). This is the candidate gene prediction task used throughout this work, and the comparison made by (Woods et al. 2013). In the second, we take a hidden gene and ask where its true disease falls among all diseases the gene is ranked for (**Fig. S7B**). This transposed view is only meaningful if scores can be compared across diseases, which the nb scheme was never designed for. A disease enters a cell of either heatmap when its median rank is at or below the

bioRxiv preprint doi: https://doi.org/10.64898/2026.10.23.761438; this version posted October 27, 2026. The copyright holder for this preprint (which was not certified by peer review) is the author/funder, who has granted bioRxiv a license to display the preprint in perpetuity. It is made available under a CC-BY 4.0 International license.

column threshold, and the total column counts the diseases whose hidden genes were surfaced at any rank. A disease must retain at least one other annotated family for any phenolog to be found, so diseases annotated to a single protein family (**Fig. S2**) cannot be evaluated, and coverage grows with k as more phenologs contribute candidates (168, 274, 322 and 332 diseases at k = 1, 5, 20 and 100).

Within a disease the two schemes are close to interchangeable (Fig. S7A). Both draw on the same candidate pool, so the total column is identical, and the counts differ by at most 24 diseases at any threshold, with the two schemes trading places from one cut-off to the next and nb marginally ahead at the looser ones. Across diseases the picture changes completely (**Fig. S7B**). At k = 20, hg places the true disease first for 18 diseases and within the top 20 for 165, whereas nb manages 0 and 12. nb also loses resolution as more phenologs are combined, consistent with its scores saturating towards one as the product in (**Eq. S1**) accumulates terms: the diseases it places within the top 20 fall from 119 at k = 1 to 4 at k = 100, while hg holds between 147 and 165 at every k. This is the property that allows hg candidates to be pooled into a single ranked list across diseases (**Fig. 3B**).

Panels C and D complement the leave-one-out analysis with a direct comparison of how the two schemes order the same candidates, computed from the complete model at k = 100 across the 414 diseases with at least four known genes. No genes are withheld here, so a disease's own annotations help find its phenologs and the absolute recall is optimistic, but both schemes are measured on identical candidate lists. hg recovers a larger fraction of each disease's known genes at every shortlist size (45% versus 39% within the top 20; **Fig. S7C**), and its per-disease area under the precision-recall curve is higher in 278 of 414 diseases (67%), by a median of 0.026 (**Fig. S7D**). Taken together, hg matches nb at ranking candidates within a disease, improves precision at the top of each list, and is the only one of the two that can be used to rank candidates across diseases.

bioRxiv preprint doi: https://doi.org/10.64898/2026.10.23.761438; this version posted October 27, 2026. The copyright holder for this preprint (which was not certified by peer review) is the author/funder, who has granted bioRxiv a license to display the preprint in perpetuity. It is made available under a CC-BY 4.0 International license.

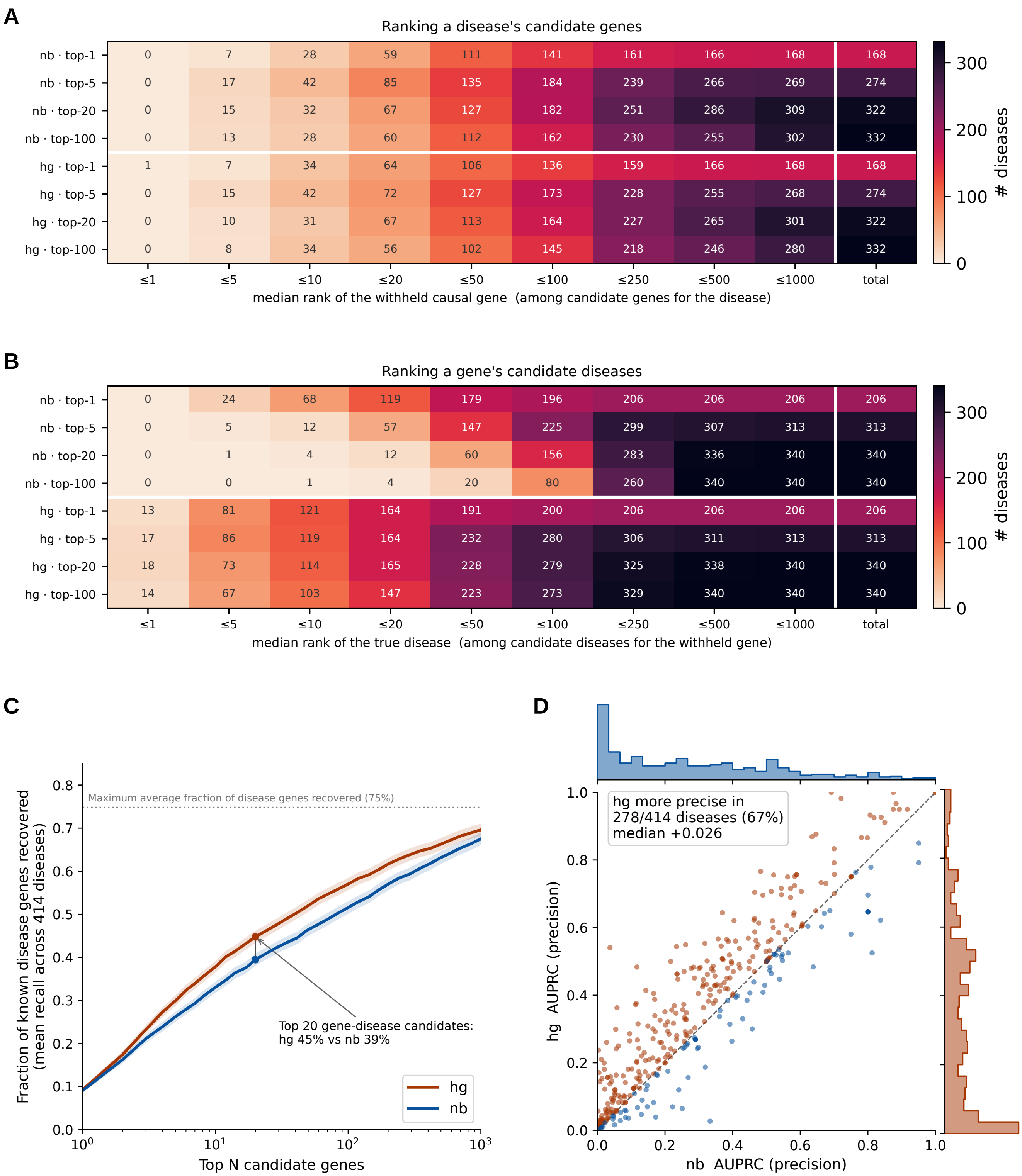


**Figure S7. Leave-one-out recovery of known gene-disease associations and the precision of the two gene ranking schemes.** (A) Ranking a disease's candidate genes. Each row is one ranking scheme (nb or hg) at one setting of k, the number of top phenologs combined per disease (1, 5, 20, 100). For each of the 2,001 PANTHER protein families carrying a human disease annotation, the family's genes are removed from the human gene-disease network, phenologs and gene rankings are recomputed, and the rank of each withheld gene is read from the ranked candidate list of every disease it was annotated to; tied scores receive the average rank. A disease's value is the

bioRxiv preprint doi: https://doi.org/10.64898/2026.10.23.761438; this version posted October 27, 2026. The copyright holder for this preprint (which was not certified by peer review) is the author/funder, who has granted bioRxiv a license to display the preprint in perpetuity. It is made available under a CC-BY 4.0 International license.

median rank over its withheld genes. Cells count the diseases whose median rank is at or below the column threshold, and the total column counts the diseases whose withheld genes appear anywhere in the candidate list. The colour scale runs from 0 to the largest total (332 in A, 340 in B). (B) Ranking a gene's candidate diseases. The same leave-one-out runs read in the other direction: the diseases ranked for each withheld gene are sorted by score and the rank of the gene's true disease is recorded, then summarized per disease and counted as in A. This direction requires scores that are comparable across diseases. (C) Recall of known disease genes by the complete model (no genes withheld; k = 100) across the 414 diseases with at least four known genes carrying an ortholog in a modeled species. Curves give the mean fraction of a disease's known genes found within its top N candidates (shading, ± 1 s.e.m.); the dotted line is the mean fraction of known genes present anywhere in the candidate lists (75%), identical for both schemes because they rank the same candidates. At N = 20, hg recovers 45% and nb 39%. (D) Per-disease area under the precision-recall curve (AUPRC) for the same 414 diseases, nb on the x axis and hg on the y axis, with marginal histograms. Orange points lie on or above the diagonal (hg at least as precise) and blue points below it. hg is more precise in 278 of 414 diseases (67%), by a median of 0.026.
